# Supplementary material for: Hyperphosphorylated PTEN exerts oncogenic properties
Source: Nat Commun. 2023 May 24;14:2983. doi: 10.1038/s41467-023-38740-x (PMC10209192; doi:10.1038/s41467-023-38740-x)

SUPPLEMENTARY INFORMATION

Hyperphosphorylated PTEN exerts oncogenic properties

Janine H van Ree[1], Karthik B Jeganathan[1], Raul O Fierro Velasco[1], Cheng Zhang[2], Ismail Can[3], Masa-kazu Hamada[1], Hu Li[2], Darren J Baker[1,3] & Jan M van Deursen[1,3,*]

[1]Department of Pediatric and Adolescent Medicine, Mayo Clinic, Rochester MN, United States, [2]Department of Molecular Pharmacology and Experimental Therapeutics, Mayo Clinic, Rochester, MN, United States, and [3]Department of Biochemistry and Molecular Biology, Mayo Clinic, Rochester MN, United States. *Correspondence: janvandeursen2@gmail.com

TABLE OF CONTENTS

Supplementary Note

Supplementary Figures 1-9

Supplementary Note

Chromosome counts on metaphase spreads of MEFs derived from *Pten*$^{S380A/A}$ and *Pten*$^{\Delta 4/\Delta 4}$ mice yielded aneuploidy rates that were comparable to those of *Pten*$^{\Delta TKV/\Delta TKV}$ MEFs (Supplementary Fig. 2a). Consistent with this, all three genotypes showed similar increases in chromosome segregation errors when movements of mitotic chromosomes were monitored by live-cell imaging (Supplementary Fig. 2b). Furthermore, key centrosome-related defects driving erroneous chromosome segregation in *Pten*$^{\Delta TKV/\Delta TKV}$ MEFs were also observed in *Pten*$^{S380A/A}$ and *Pten*$^{\Delta 4/\Delta 4}$ MEFs (Supplementary Fig. 2c-e). This included impairments in recruiting the mitotic motor protein EG5 to centrosomes, movement of duplicated centrosomes to opposite poles during bipolar spindle formation, and perpendicular mitotic spindle formation. Thus, lack of PTEN C-tail phosphorylation at S380 resulted in mitotic phenotypes that are reminiscent of those reported for PTEN without the C-terminal PDZ binding domain. Collectively, these data indicate that mitotic defects caused by centrosomal PTEN deficiency observed in *Pten*$^{S380A/A}$ and *Pten*$^{\Delta 4/\Delta 4}$ cells could be due to PTEN^{S380A} and PTEN$^{\Delta 4}$ instability rather than support a model in which S380 phosphorylation is a requirement for DLG1-mediated centrosomal accumulation of PTEN.

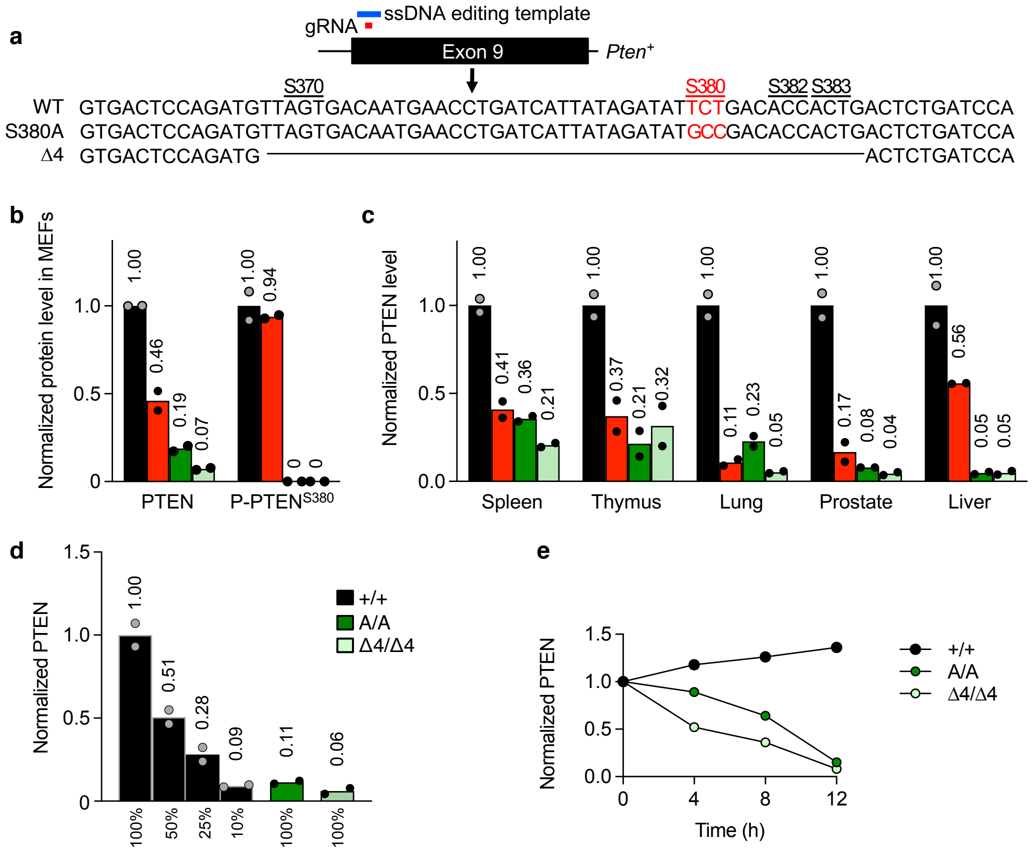

Supplementary Figure 1. PTEN^{S380A} and PTEN$^{\Delta 4}$ are expressed at subnormal levels. a Targeting design of CRISPR-CAS9-generated mutations in *Pten*^{S380A} and *Pten*$^{\Delta 4}$. **b-d** Quantitation of WB signal depicted in **Fig. 1b**-**e**. Protein level is normalized to Ponceau S. Numbers above the bars represent the average normalized value compared to +/+. **e** Quantitation of WB signal depicted in **Fig. 1f**. PTEN protein level is normalized to Tubulin and graphed in time. Source data are provided as a Source Data file.

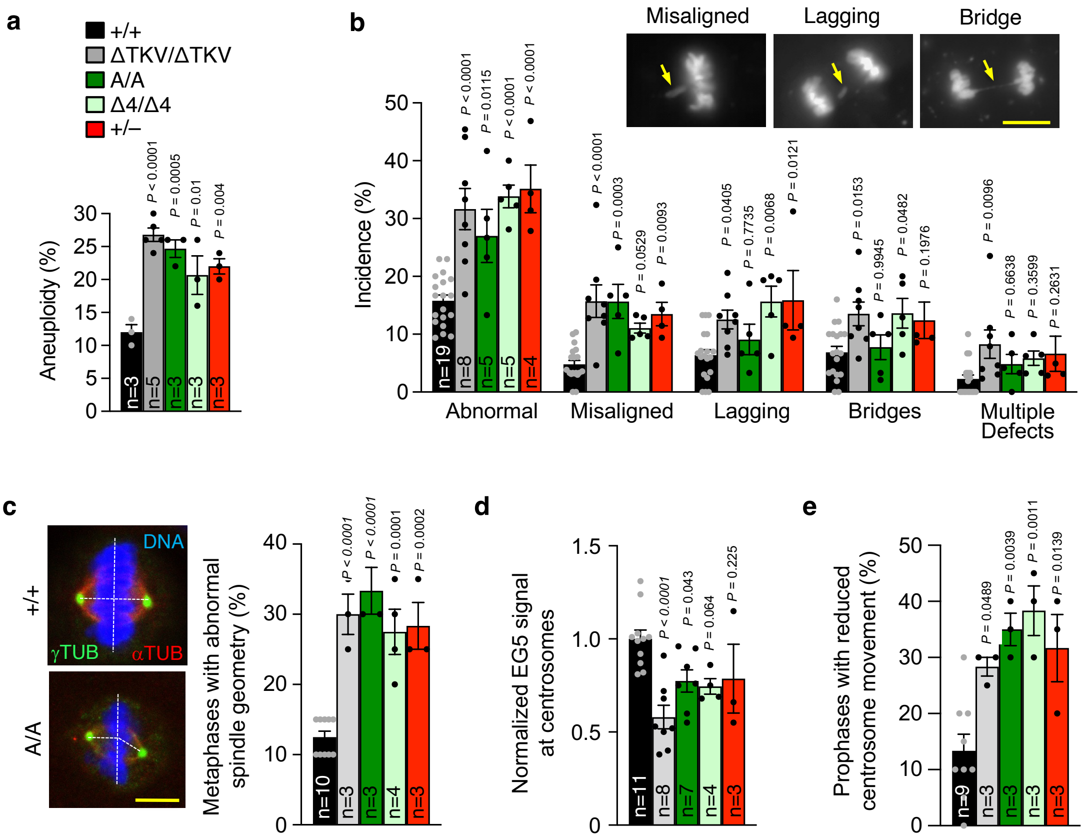

Supplementary Figure 2. PTEN C-tail phosphorylation is required for proper chromosome segregation. a Chromosome counts on P5 MEFs (50 spreads per line). A/A, *Pten*$^{S380A/A}$. **b** MEFs of indicated genotypes monitored for chromosome segregation defects by live-cell imaging (≥28 cells per line analyzed), with images showing examples of defects. Cells are transduced with lentivirus TSIN-H2B-mRFP to visualize DNA. Scale bar, 10 µm. **c** Images of MEFs in metaphase immunostained for α-tubulin (red) and γ-tubulin (green), with incidence of MEFs with asymmetrical metaphase spindles. **d** Quantification of immunostained EG5 signal at astral microtubules and centrosomes in prophase. **e** Prophases with reduced centrosome movement. 20 cells per MEF line were analyzed in **c**-**e**. n is the number of individual MEF lines analyzed. Statistical significance was assessed by ordinary one-way ANOVA, followed by Dunnett's multiple comparisons test compared to +/+. P-values are indicated above the bars. Source data are provided as a Source Data file.

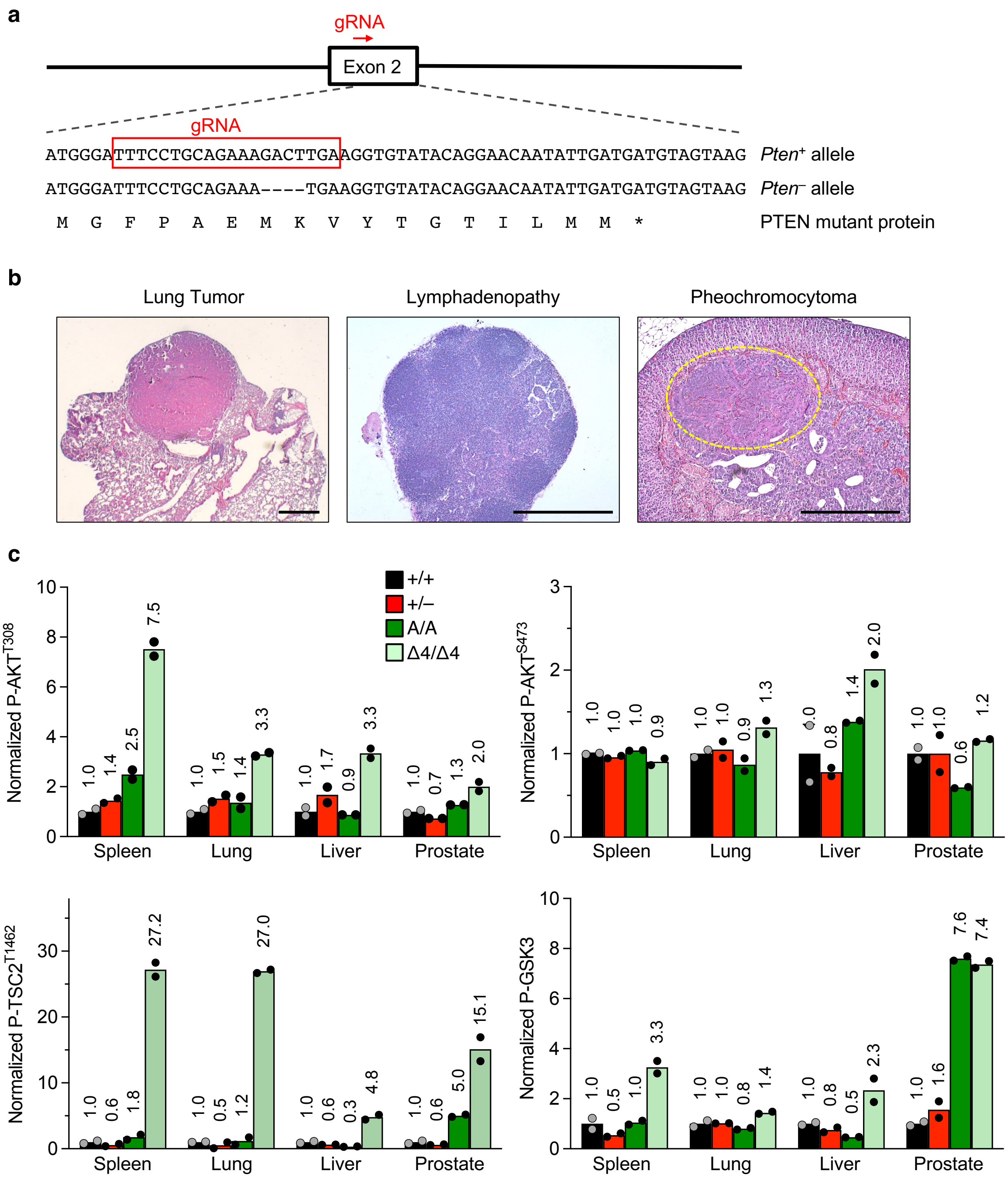

Supplementary Figure 3. Generation of *Pten*$^{+/-}$ mice. a Targeting design of CRISPR-CAS9-generated knockout mutation of *Pten* to generate *Pten*$^{+/-}$ on an FVB background. A four-nucleotide deletion in exon 2 gives a frameshift that results in a premature stop codon (marked with *). DNA and protein sequence of part of exon 2 is indicated. **b** H&E staining of a lung tumor, lymphadenopathy and pheochromocytoma as described in Figure 2b. Scale bar is 500 µm. **c** Quantitation of WB signal depicted in **Fig. 2f**. Protein level for P-AKT^{T308} and P-AKT^{S473} is normalized to total AKT, while protein level for P-TSC2^{T1462} and P-GSK3α^{S21}β^{S9} is normalized to Ponceau S. Numbers above the bars represent the mean normalized value compared to +/+. Source data are provided as a Source Data file.

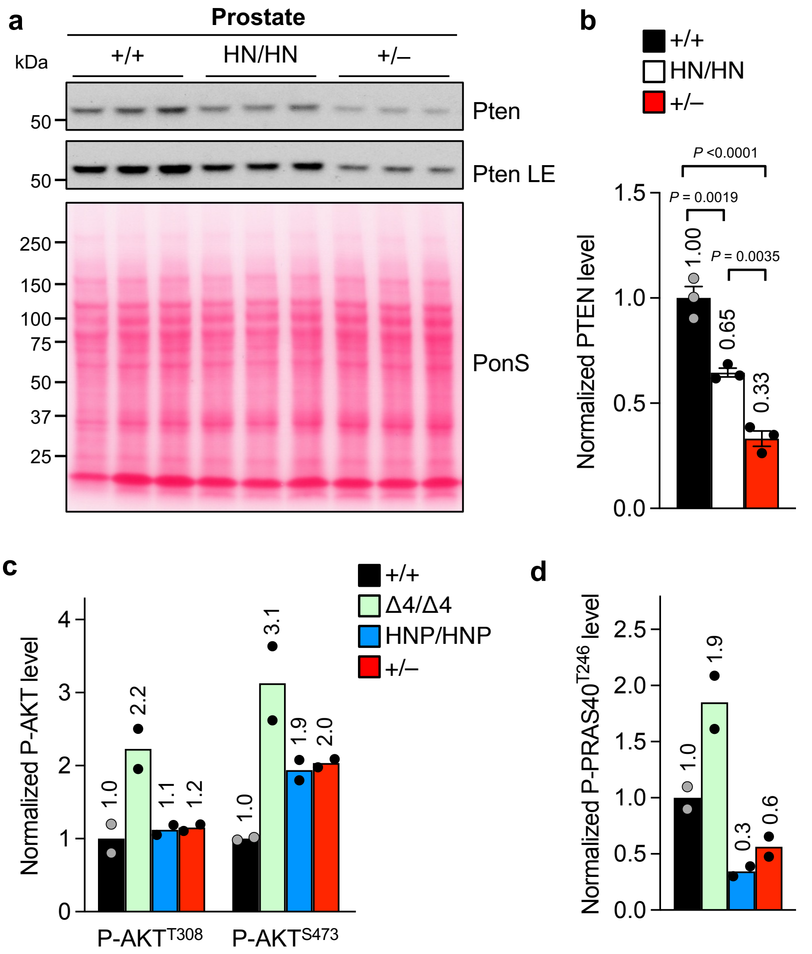

Supplementary Figure 4. Hypomorphic *Pten*^{HN} mice have slightly reduced PTEN level. a WB of prostate lysates harvested at 2 months, probed for PTEN. LE, long exposure; PonS, Ponceau S; HN/HN, *Pten*$^{HN/HN}$. Blot is representative for at least 3 samples per genotype. **b** Quantitation of WB signal in **a**. PTEN protein level is normalized to Ponceau S and presented as mean value +/– SEM**.** Statistical significance was assessed by ordinary one-way ANOVA, followed by Tukey's multiple comparisons test **c**,**d** Quantitation of WB signal in **Fig. 3b**. Protein level is normalized to total AKT in **c** and Ponceau S in **d**. Numbers above the bars represent the average normalized value compared to +/+. Source data are provided as a Source Data file.

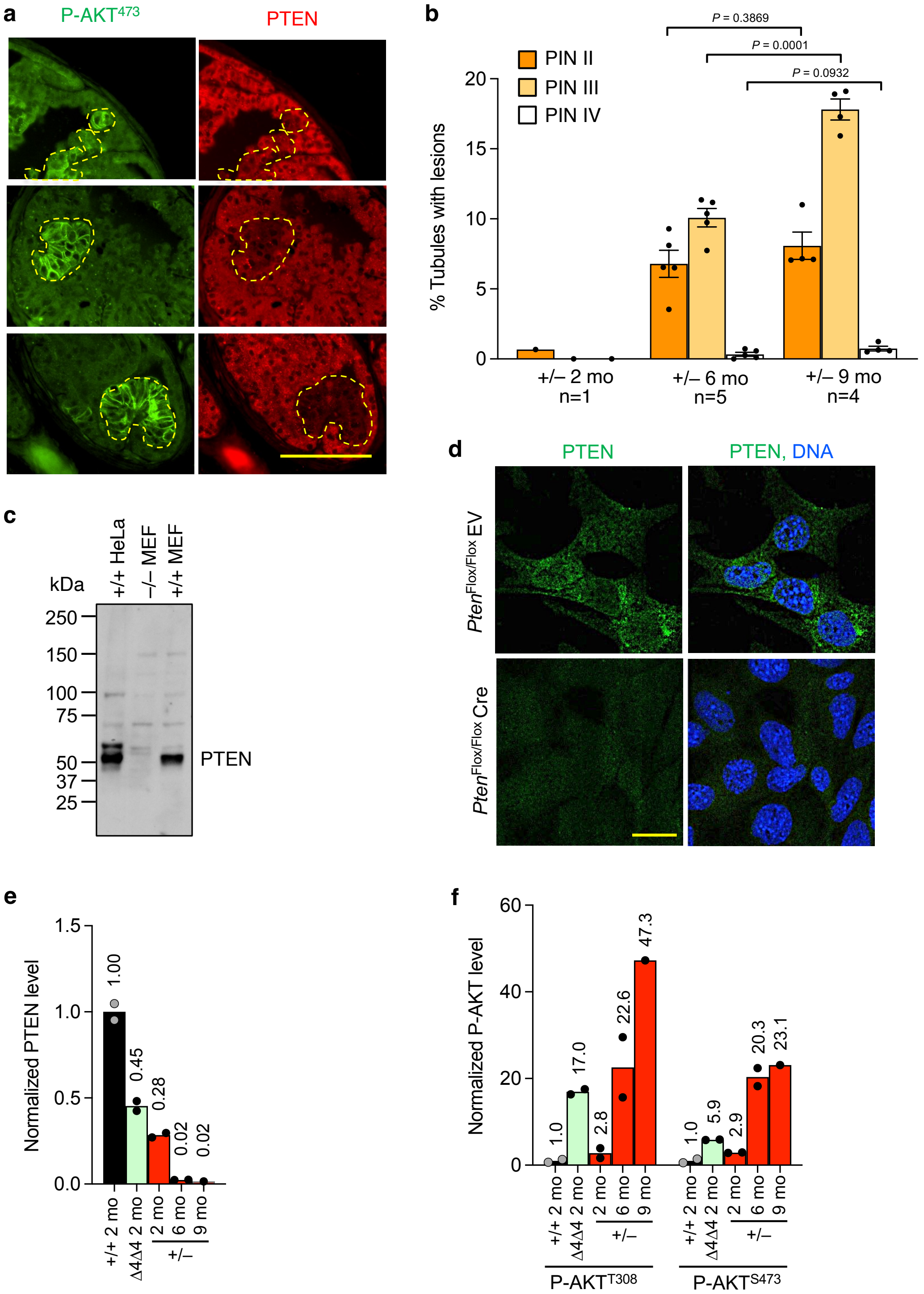

Supplementary Figure 5. Loss of catalytic activity in *Pten*$^{+/-}$ PIN lesions. a Immunostaining of consecutive sections of small PIN lesions in *Pten*$^{+/-}$ mice stained for PTEN and P-AKT^{S473} (Scale bar is 100 µm). **b** Percentage of prostate tubules with indicated PIN lesions in *Pten*$^{+/-}$ mice at 2, 6 and 9 months. n, number of prostates. Samples at 6 months are the same as those depicted in **Fig. 3d**. Data are presented as mean +/- SEM. Statistical significance was assessed by two-tailed unpaired t-test between 6- and 9-month-old mice. **c** WB testing of home-made rabbit polyclonal antibody against mouse PTEN (n=1). Immortalized *Pten*$^{Flox/Flox}$ MEFs with stably transfected lentivirus TSIN-empty vector (+/+) or TSIN-Cre (–/–) were used to test reactivity and specificity against mouse cells, while HeLa were used to test for reactivity against human cells. **d** IF on 3% PFA-fixed MEFs as in (c), immuno-labeled for PTEN, and DNA visualized by Hoechst (n=1). Scale bar is 20 µm. **e**,**f** Quantitation of WB signal in **Fig. 4d**. Protein level is normalized to Ponceau S in **e** and total AKT in **f**. Numbers above the bars represent the average normalized value compared to +/+. Source data are provided as a Source Data file.

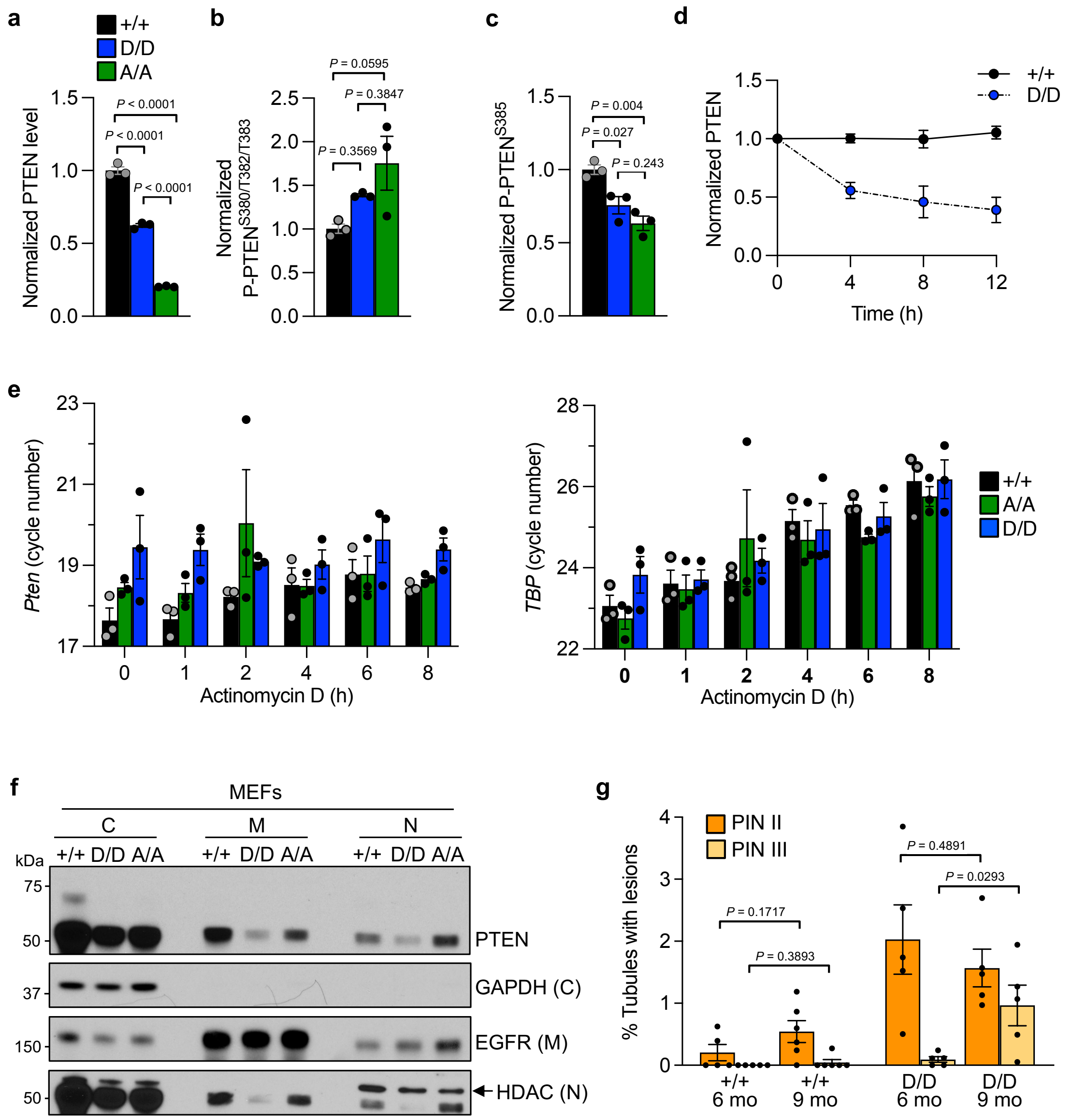

Supplementary Figure 6. PTEN^{S380D} is unstable. a-c Quantitation of WB signal depicted in **Fig. 5a** (n=1 experiment with 3 individual samples per genotype). Protein level is normalized to Ponceau S in **a** and to PTEN in **b** and **c**. **d** Quantitation of WB signal depicted in **Fig. 5c**. PTEN protein level is normalized to Tubulin and graphed in time. N=3 independent experiments. **e** qRT-PCR of *Pten* expression in MEFs of specified genotypes after treatment with Actinomycin D for indicated time. Data are mean cycle number for *Pten* or *TBP* (normalizer) ± SEM of triplicate measurements (n=3 independent cell lines for each genotype). **f** Cell fractionation of MEFs of specified genotypes. Indicated fractions (C, cytoplasm; M, membrane; N, nuclear) were blotted and probed for PTEN and compartment-specific proteins. n = 3 independent fractionation experiments. **g** Percentage of prostate tubules with indicated PIN lesions at 6 and 9 months. +/+ and D/D at 6 months are the same samples as depicted in **Fig. 5f**. n=6 for +/+ prostates at 9 months and n=5 for all other prostates per genotype. Data are presented as mean values +/- SEM. Statistical significance in **a**-**e** was assessed by ordinary one-way ANOVA, followed by Tukey's multiple comparisons test and in **g** by two-tailed unpaired t test between 6- and 9-month-old mice. Source data are provided as a Source Data file.

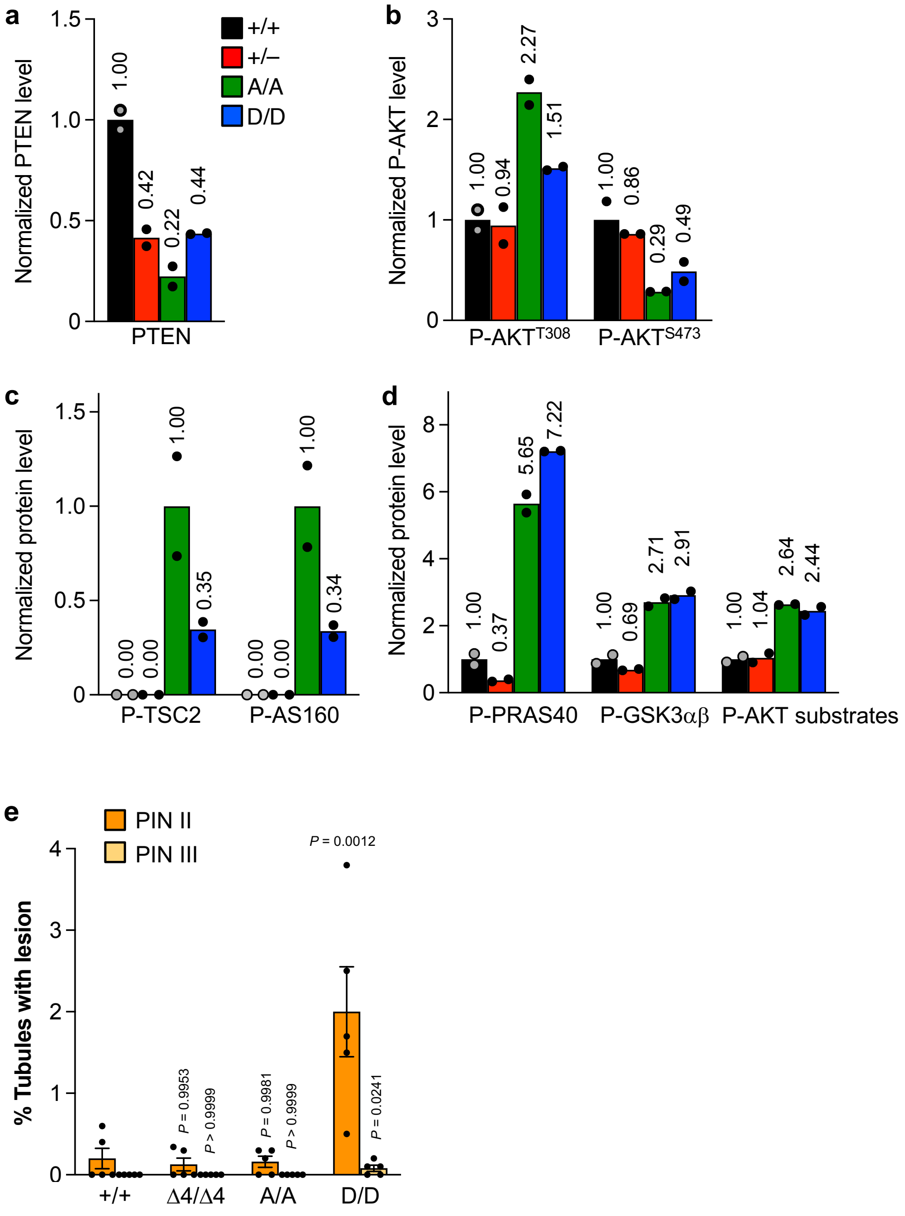

Supplementary Figure 7. Hyperactive AKT signaling in S380 mutants. a-d Quantitation of WB signal in **Fig. 5e**. Protein level in **a**, **c** and **d** is normalized to Ponceau S and to total AKT in **b**. Numbers above the bars represent the average normalized value compared to +/+ in **a**, **b** and **d**, while in **c** it represents the average normalized value compared to PTEN^{S380A}. **e** Percentage of prostate tubules with indicated PIN lesions at 6 months. +/+, A/A and D/D are the same samples as depicted in **Fig. 5f**. N=5 prostates per genotype. Data are presented as mean values +/- SEM. Statistical significance was assessed by ordinary one-way ANOVA, followed by Dunnett's multiple comparisons test compared to +/+. Source data are provided as a Source Data file.

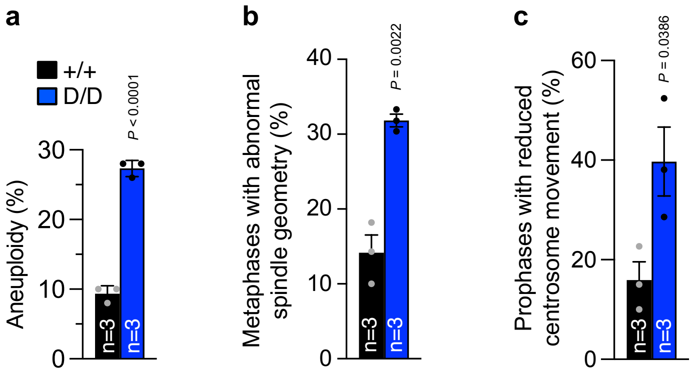

Supplementary Figure 8. Mitotic abnormalities in *Pten*^{S380D} MEFs. a Chromosome counts on P5 MEFs (50 spreads per line). **b** Incidence of MEFs with asymmetrical metaphase spindles. **c** Prophases with reduced centrosome movement. At least 20 cells per MEF line were analyzed in **b**-**c**. n is the number of individual MEF lines analyzed. Data are presented as mean +/– SEM. Statistical significance was assessed by two-tailed unpaired t test. Source data are provided as a Source Data file.

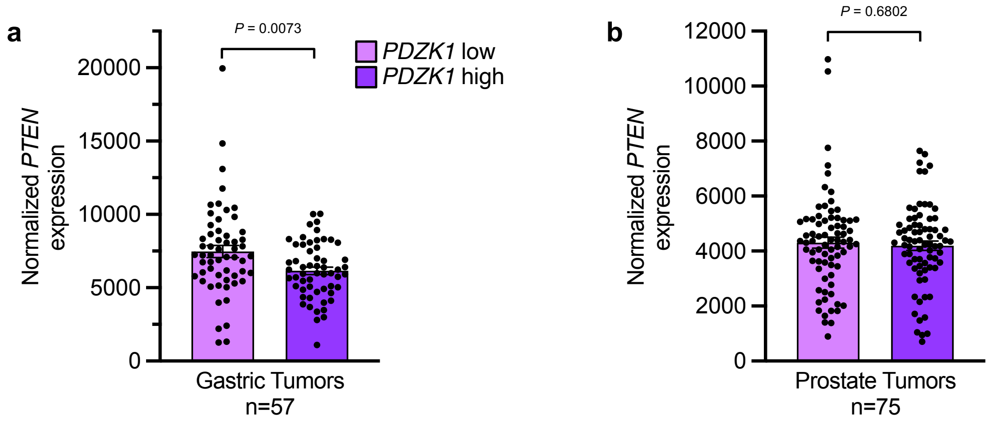

Supplementary Figure 9. *PTEN* is expressed in gastric and prostate cancers with low levels of *PDZK1*. a Quantitation of normalized expression of *PTEN* in samples with low *PDZK1* expression (bottom 15%; n = 57 cases) and high *PDZK1* expression (top 15%; n = 57 cases) in TGCA-STAD gastric cancers. **(b)** Same as in **a** but now for TGCA-PRAD prostate cancers. Top and bottom 15% each have n=75 cases. Data are presented as mean +/– SEM. Statistical significance was assessed by two-tailed unpaired t test. Source data are provided as a Source Data file.

Uncropped blots:

Supplementary Fig. 4a

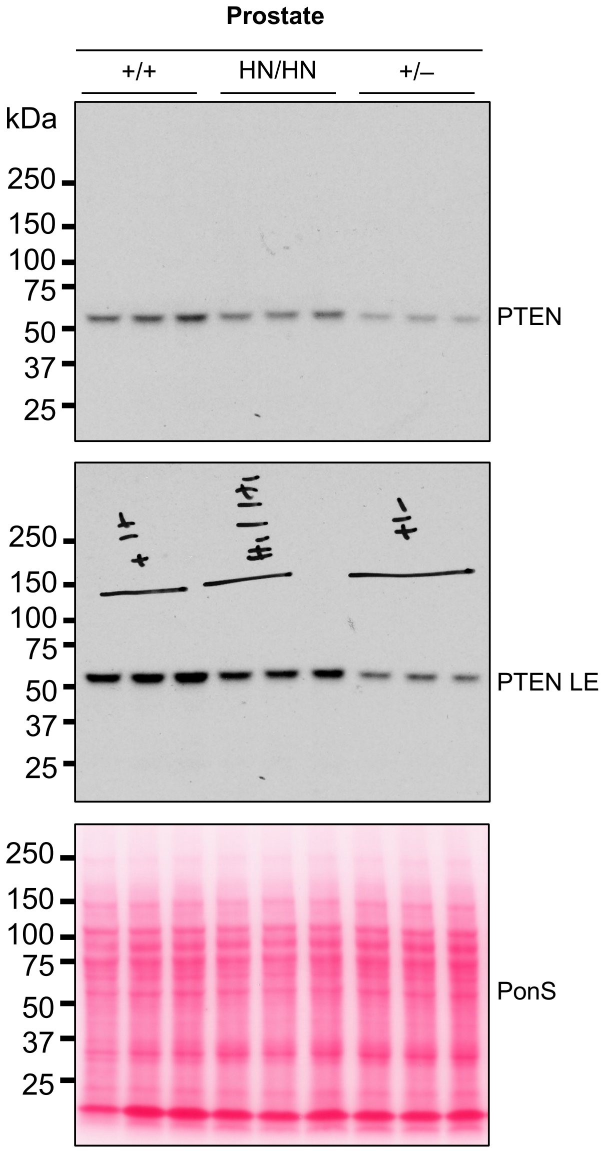

Uncropped blots:

Supplementary Fig. 6f

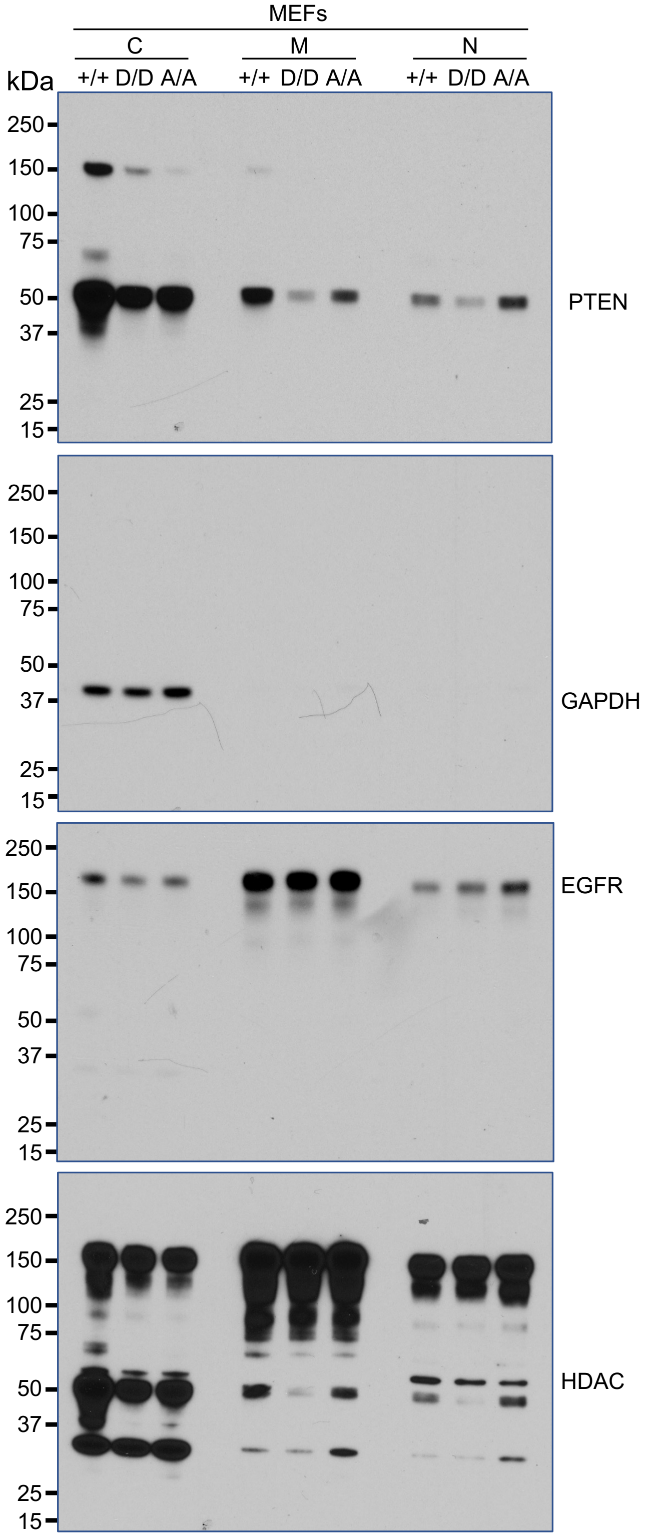

Supplement: Supplementary file 1 — Supplementary Information [file 41467_2023_38740_MOESM1_ESM.pdf]
