# Supplementary material for: Hyperphosphorylated PTEN exerts oncogenic properties
Source: Nat Commun. 2023 May 24;14:2983. doi: 10.1038/s41467-023-38740-x (PMC10209192; doi:10.1038/s41467-023-38740-x)
Supplement: Supplementary file 2 — Reporting Summary [file 41467_2023_38740_MOESM2_ESM.pdf]

nature portfolio

Corresponding author(s): van Deursen

Last updated by author(s): 04-16-2023

Reporting Summary

Nature Portfolio wishes to improve the reproducibility of the work that we publish. This form provides structure for consistency and transparency in reporting. For further information on Nature Portfolio policies, see our Editorial Policies and the Editorial Policy Checklist.

Statistics

For all statistical analyses, confirm that the following items are present in the figure legend, table legend, main text, or Methods section.

n/a	Confirmed	
☐	☒	The exact sample size (*n*) for each experimental group/condition, given as a discrete number and unit of measurement
☐	☒	A statement on whether measurements were taken from distinct samples or whether the same sample was measured repeatedly
☐	☒	The statistical test(s) used AND whether they are one- or two-sided<br>*Only common tests should be described solely by name; describe more complex techniques in the Methods section.*
☒	☐	A description of all covariates tested
☐	☒	A description of any assumptions or corrections, such as tests of normality and adjustment for multiple comparisons
☐	☒	A full description of the statistical parameters including central tendency (e.g. means) or other basic estimates (e.g. regression coefficient) AND variation (e.g. standard deviation) or associated estimates of uncertainty (e.g. confidence intervals)
☐	☒	For null hypothesis testing, the test statistic (e.g. *F*, *t*, *r*) with confidence intervals, effect sizes, degrees of freedom and *P* value noted<br>*Give P values as exact values whenever suitable.*
☒	☐	For Bayesian analysis, information on the choice of priors and Markov chain Monte Carlo settings
☒	☐	For hierarchical and complex designs, identification of the appropriate level for tests and full reporting of outcomes
☒	☐	Estimates of effect sizes (e.g. Cohen's *d*, Pearson's *r*), indicating how they were calculated

Our web collection on statistics for biologists contains articles on many of the points above.

Software and code

Policy information about availability of computer code

Data collection	Zeiss ZEN Black v.14.0.24.201 software was used to acquire images of immunofluorescence experiments on a Zeiss LSM880 confocal laser-scanning microscope and Zen Blue to acquire images of live cells on an Axiovision Observer microscope (Zeiss). Olympus cellSens 2.3 was used to acquire images of fluorescence experiments and H&E stainings on an Olympus BX63 microscope and to acquire low magnification images of tissue on an Olympus SZX16 stereo microscope.<br>RNA sequencing: Flow cells were sequenced as 100x2 paired-end reads on an Illumina HiSeq 4000 using HiSeq 3000/4000 sequencing kit and HCS v.3.3.20 collection software. Base-calling was performed using Illumina's RTA v.2.5.2 software.
Data analysis	GraphPad Prism 9.3.1 was used for statistical assessments.<br>ImageJ 1.52a was used for immunoblot densitometry.<br>Zeiss ZEN Black software v.14.0.24.201 was used to take and export immunofluorescence images, and to measure spindle geometry and centrosome movement.<br>Fastqfiles of paired-end RNA-Seq reads were aligned with Tophat v.2.0.1467 against the UCSC reference genome mm10 (http://genome.ucsc.edu/cgi-bin/hgGateway?db=mm10) using Bowtie v.2 2.2.6 with default settings.<br>Gene level counts from read pairs were obtained using FeatureCounts v.1.4.6 from the SubRead package68 and gene models from the UCSC mm10 annotation.<br>Differential expression analysis was performed using R package DESeq2 v.1.10.1 after removing genes with average raw counts <10.<br>Morpheus (Broad Institute) was used to generate heatmaps.<br>Overrepresentation analysis for transcription regulatory targets of individual TFs was performed using the Fisher's exact test method for selected gene lists against the mouse gene sets from ENCODE and MSigDB collections

For over-representation analysis on gastric and prostate cancers in the TCGA database, we obtained transcriptomic data from the GDC portal by using R package TCGABiolinks as raw counts.

For manuscripts utilizing custom algorithms or software that are central to the research but not yet described in published literature, software must be made available to editors and reviewers. We strongly encourage code deposition in a community repository (e.g. GitHub). See the Nature Portfolio guidelines for submitting code & software for further information.

Data

Policy information about availability of data

All manuscripts must include a data availability statement. This statement should provide the following information, where applicable:

- Accession codes, unique identifiers, or web links for publicly available datasets
- A description of any restrictions on data availability
- For clinical datasets or third party data, please ensure that the statement adheres to our policy

The RNA-seq data generated in this study have been deposited in the Gene Expression Omni-bus database under accession number GSE206157 https://www.ncbi.nlm.nih.gov/geo/query/acc.cgi?acc=GSE206157.

References to public databases used are as follows:
UCSC reference genome mm10 (http://genome.ucsc.edu/cgi-bin/hgGateway?db=mm10).
ENCODE (https://www.encodeproject.org/)
MSigDB (https://www.gsea-msigdb.org/gsea/msigdb/mouse/collections.jsp)
TCGA STAD and TCGA PRAD (https://bioconductor.org/packages/release/bioc/html/TCGAbiolinks.html).

Source data are provided with this paper.

Research involving human participants, their data, or biological material

Policy information about studies with human participants or human data. See also policy information about sex, gender (identity/presentation), and sexual orientation and race, ethnicity and racism.

Reporting on sex and gender	The only human data used in this study relate to RNA sequencing reads in publicly available databases TCGA-STAD (stomach adenocarcinoma) and TCGA-PRAD (prostate adenocarcinoma). Sex has not been considered in the study design.
Reporting on race, ethnicity, or other socially relevant groupings	N/A
Population characteristics	N/A
Recruitment	N/A
Ethics oversight	N/A

Note that full information on the approval of the study protocol must also be provided in the manuscript.

Field-specific reporting

Please select the one below that is the best fit for your research. If you are not sure, read the appropriate sections before making your selection.

[x] Life sciences [ ] Behavioural & social sciences [ ] Ecological, evolutionary & environmental sciences

For a reference copy of the document with all sections, see nature.com/documents/nr-reporting-summary-flat.pdf

Life sciences study design

All studies must disclose on these points even when the disclosure is negative.

Sample size	Sample sizes were based on previously published experiments where differences were observed. We note that no power calculations were used. The following previously published studies guided our samples sizes:<br>-) van Ree et al. 2016 (PMID 27240320) Pten regulates spindle pole movement through Dlg1-mediated recruitment of Eg5 to centrosomes.<br>-) Baker et al. 2004 (PMID 15208629) BubR1 insufficiency causes early onset of aging-associated phenotypes and infertility in mice.<br>-) Baker et al. 2009 (PMID 19962666) Whole chromosome instability caused by Bub1 insufficiency drives tumorigenesis through tumor suppressor gene loss of heterozygosity.<br>-) Limzerwala et al 2020 (PMID 34841254) FoxM1 insufficiency hyperactivates Ect2-RhoA-mDia1 signaling to drive cancer.

Data exclusions	No samples were excluded from the study.
Replication	Number of replicates is described in figures or figure legends and it is indicated whether they were technical or biological replicates.
Randomization	Randomization was not applicable to any experiments performed due to lack of drug treatments or interventions as well as experiments needing clearly defined groups of mice or cell lines based on genotype.
Blinding	Blinding to allocation during experiments was not applicable to any of the experiments. Investigators were not blinded to assessment outcome during experimentation, as they were performed by the same researcher for consistency and feasibility.

Reporting for specific materials, systems and methods

We require information from authors about some types of materials, experimental systems and methods used in many studies. Here, indicate whether each material, system or method listed is relevant to your study. If you are not sure if a list item applies to your research, read the appropriate section before selecting a response.

Materials & experimental systems

n/a	Involved in the study	
☐	☒	Antibodies
☐	☒	Eukaryotic cell lines
☒	☐	Palaeontology and archaeology
☐	☒	Animals and other organisms
☒	☐	Clinical data
☒	☐	Dual use research of concern
☒	☐	Plants

Methods

n/a	Involved in the study	
☒	☐	ChIP-seq
☒	☐	Flow cytometry
☒	☐	MRI-based neuroimaging

Antibodies

Antibodies used

All antibodies used are listed in the Methods section.

Co-immunoprecipitation: rabbit anti-PTEN (Cell Signaling #9559), 15 µl per IP
Co-immunoprecipitation: rabbit IgG (Southern Biotech #0111-01), 1 µl per IP
Co-immunoprecipitation: rabbit anti-C-terminal hPTEN (51-2400, Invitrogen), 1 µg per IP
Co-imunoprecipitation: rabbit anti-Red Fluorescent Protein (anti-RFP) (MA5-15257, Invitrogen), 1 µg per IP

Western blot: goat anti-mouse, HRP-conjugated (Jackson Immunoresearch; #115-035-146; 1:10,000)
Western blot: goat anti-rabbit, HRP-conjugated (Jackson Immunoresearch; #111-035-003; 1:10,000)

Western blot: rabbit anti-PTEN(9559, Cell Signaling Technology, 1:2,000)
Western blot: rabbit anti human PTEN (Cascade, ABM-2052, 1:4,000)
Western blot: rabbit anti-P-PTEN-S380 (9551, Cell Signaling Technology, 1:1,000)
Western blot: rabbit anti-P-PTEN-S380/T382/T383 (9549, Cell Signaling Technology, 1:1,000)
Western blot: rabbit anti-P-PTEN-S385 (07-890-I, Sigma-Aldrich, 1:1,000)
Western blot: rabbit anti-Tubulin (2125, Cell Signaling Technology, 1:2,000)
Western blot: rabbit anti-EGFR (71655S, Cell Signaling Technology, 1: 1,000)
Western blot: rabbit anti-HDAC (Ab7028, Abcam, 1:1,000)
Western blot: rabbit anti-Akt (9272 Cell Signaling Technology, 1:1,000)
Western blot: rabbit anti-P-AKT-T308 (2965, Cell Signaling Technology, 1:1,000)

Western blot: rabbit anti-P-AKT-S473 (9271, Cell Signaling Technology, 1:1,000)
Western blot: rabbit-anti-P-TSC2-T1462 (3617, Cell Signaling Technology, 1:500)
Western blot: rabbit-anti-P-GSK3aS21/bS9 (9331, Cell Signaling Technology, 1;1,000)
Western blot: rabbit-anti-P-PRAS40-T246 (2997, Cell Signaling Technology, 1:2,000)
Western blot: rabbit-anti-P-AS160-T642 (8881, Cell Signaling Technology, 1:500)
Western blot: rabbit-anti-P-AKT substrates (9614, Cell Signaling Technology, 1:500)
Western blot: rabbit-anti-GAPDH (3683, Cell Signaling Technology, 1:1,000)
Western blot: mouse-anti-b-catenin (610153, BD, 1:2,000).

Immunofluorescence: goat anti-mouse Alexa Fluor 488 (Invitrogen, A11001; 1:250)
Immunofluorescence: goat anti-rabbit Alexa Fluor 488 (Invitrogen, A11008; 1:250)
Immunofluorescence: goat anti-rabbit Alexa Fluor 594 (Invitrogen, A11012; 1:250)
Immunofluorescence: goat anti-mouse Alexa Fluor 594 (Invitrogen, A11005; 1:250)
Immunofluorescence: goat anti-mouse IgG1 Alexa Fluor 488 (Invitrogen, A21121; 1:250)

Immunofluorescence: rabbit anti-Eg5 (1:100, TA301478, Origene)
Immunofluorescence: mouse anti-a-tubulin (1:1,000, T9026, Sigma)
Immunofluorescence: mouse or rabbit anti-g-tubulin (1:300, T6557 or T5192, Sigma)
Immunofluorescence: rabbit anti-P-AKTS473 (1:100, 4060, Cell signaling Technology)
Immunofluorescence: rabbit anti-P- AKTS473-Alexa Fluor 488 conjugate (1:50, 4071, Cell Signaling Technology)
Immunofluorescence: mouse anti-bcatenin (1:200, 610153, BD)
Immunofluorescence: rabbit anti-p-histone H3S10 (1:1,000, 06-570, Millipore)
Immunofluorescence: rabbit anti-PTEN (1:50, 2551, home-made).

Validation

The rabbit anti-PTEN antibody used for tissue IF was validated in house by Western blot and IF using tissues or cell lines derived from genetically engineered mouse strains.

Commercially available antibodies were validated by the manufacturer and the published literature utilizing these antibodies. Please find validation notes and citations for the utilized antibodies, directly on the websites of the manufacturers below.

rabbit anti-PTEN (9559, Cell Signaling Technology)
https://www.cellsignal.com/products/primary-antibodies/pten-138g6-rabbit-mab/9559

rabbit IgG (0111-01, Southern Biotech)
https://www.southernbiotech.com/rabbit-igg-unlb-0111-01

rabbit anti-C-terminal hPTEN (51-2400, Invitrogen)
https://www.thermofisher.com/antibody/product/PTEN-Antibody-Polyclonal/51-2400

rabbit anti-Red Fluorescent Protein (anti-RFP) (MA5-15257, Invitrogen)
https://www.thermofisher.com/antibody/product/RFP-Antibody-clone-RF5R-Monoclonal/MA5-15257

rabbit anti human PTEN (ABM-2052, Cascade)
http://www.cascadebioscience.com/pages/Products_and_Parts/PTEN/PTEN_ABM-2052.html

rabbit anti-P-PTEN-S380 (9551, Cell Signaling Technology)
https://www.cellsignal.com/products/primary-antibodies/phospho-pten-ser380-antibody/9551

rabbit anti-P-PTEN-S380/T382/T383 (9549, Cell Signaling Technology)
https://www.cellsignal.com/products/primary-antibodies/phospho-pten-ser380-thr382-383-44a7-rabbit-mab/9549

rabbit anti-P-PTEN-S385 (07-890-I, Sigma-Aldrich)
https://www.sigmaaldrich.com/US/en/product/mm/07890i

rabbit anti-Tubulin (2125, Cell Signaling Technology)
https://www.cellsignal.com/products/primary-antibodies/a-tubulin-11h10-rabbit-mab/2125

rabbit anti-EGFR (71655, Cell Signaling Technology)
https://www.cellsignal.com/products/primary-antibodies/egf-receptor-d1p9c-rabbit-mab/71655?site-search-type=Products&N=4294956287&Ntt=71655s&fromPage=plp&_requestid=702997

rabbit anti-HDAC (Ab7028, Abcam)
https://www.abcam.com/products/primary-antibodies/hdac1-antibody-ab7028.html

rabbit anti-Akt (9272 Cell Signaling Technology)
https://www.cellsignal.com/products/primary-antibodies/akt-antibody/9272

rabbit anti-P-AKT-T308 (2965, Cell Signaling Technology)
https://www.cellsignal.com/products/primary-antibodies/phospho-akt-thr308-c31e5e-rabbit-mab/2965

rabbit anti-P-AKT-S473 (9271, Cell Signaling Technology)
https://www.cellsignal.com/products/primary-antibodies/phospho-akt-thr308-c31e5e-rabbit-mab/2965

rabbit-anti-P-TSC2-T1462 (3617, Cell Signaling Technology)
https://www.cellsignal.com/products/primary-antibodies/phospho-tuberin-tsc2-thr1462-5b12-rabbit-mab/3617

rabbit-anti-P-GSK3aS21/bS9 (9331, Cell Signaling Technology)
https://www.cellsignal.com/products/primary-antibodies/phospho-gsk-3a-b-ser21-9-antibody/9331

rabbit-anti-P-PRAS40-T246 (2997, Cell Signaling Technology)
https://www.cellsignal.com/products/primary-antibodies/phospho-pras40-thr246-c77d7-rabbit-mab/2997

rabbit-anti-P-AS160-T642 (8881, Cell Signaling Technology)
https://www.cellsignal.com/products/primary-antibodies/phospho-as160-thr642-d27e6-rabbit-mab/8881

rabbit-anti-P-AKT substrates (9614, Cell Signaling Technology)
https://www.cellsignal.com/products/primary-antibodies/phospho-akt-substrate-rxxs-t-110b7e-rabbit-mab/9614

rabbit-anti-GAPDH (3683, Cell Signaling Technology)
https://www.cellsignal.com/products/antibody-conjugates/gapdh-14c10-rabbit-mab-hrp-conjugate/3683

mouse-anti-b-catenin (610153, BD).
https://www.bdbiosciences.com/en-ca/products/reagents/microscopy-imaging-reagents/immunofluorescence-reagents/purified-mouse-anti-catenin.610153

rabbit anti-Eg5 (TA301478, Origene)
https://www.origene.com/catalog/antibodies/primary-antibodies/ta301478/eg5-kif11-rabbit-polyclonal-antibody

mouse anti-a-tubulin (T9026, Sigma)
https://www.sigmaaldrich.com/US/en/product/sigma/t9026

mouse anti-g-tubulin (T6557, Sigma)
https://www.sigmaaldrich.com/US/en/product/sigma/t6557

rabbit anti-g-tubulin (T5192, Sigma)
https://www.sigmaaldrich.com/US/en/product/sigma/t5192

rabbit anti-P-AKTS473 (4060, Cell signaling Technology)
https://www.cellsignal.com/products/primary-antibodies/phospho-akt-ser473-d9e-xp-rabbit-mab/4060

anti-P- AKTS473-Alexa Fluor 488 conjugate (4071, Cell Signaling Technology)
https://www.cellsignal.com/products/antibody-conjugates/phospho-akt-ser473-d9e-xp-rabbit-mab-alexa-fluor-488-conjugate/4071

rabbit anti-p-histone H3S10 (06-570, Millipore)
https://www.sigmaaldrich.com/US/en/product/sigma/h0412?gclid=EAIaIQobChMIz7zR9vis_gIVMhitBh1_QwZ0EAAYASAAEgIO9fD_BwE&gclsrc=aw.ds

Eukaryotic cell lines

Policy information about cell lines and Sex and Gender in Research

Cell line source(s)	Primary MEFs, mouse embryonic fibroblasts, were generated within the laboratory from genetically modified or unmodified mice.<br>HeLa cells have been in the lab a long time but came originally from ATCC (CCL-2).
Authentication	PCR-based genotyping was used for cell identity authentication.
Mycoplasma contamination	All MEF cultures were primary cultures that were used at early passage. They were not tested for mycoplasma
Commonly misidentified lines (See ICLAC register)	No commonly misidentified cell lines were used in this study.

Animals and other research organisms

Policy information about studies involving animals; ARRIVE guidelines recommended for reporting animal research, and Sex and Gender in Research

Laboratory animals	Mice were used for generation of mouse embryonic fibroblasts as described in the methods section. MEFs were generated from embryos at embryonic day E13.5. The sex of embryos was not determined. Mice were maintained on a C57BL/6 or a FVB genetic background. All mice were housed in a specific-pathogen-free (SPF) barrier environment with ad libitum access to food and water, 12-hour light and dark cycles, temperature between 68-79oF (average of 71 oF) and humidity between 30-70% (average of 45%).
Wild animals	This study did not involve wild animals.
Reporting on sex	This study was focused on mouse prostate and therefore used male mice.

Field-collected samples	No samples were collected from the field.
Ethics oversight	Experimental procedures involving laboratory mice were reviewed and approved by the Institutional Animal Care and Use Committee of the Mayo Clinic.

Note that full information on the approval of the study protocol must also be provided in the manuscript.
